# Sodium butyrate alleviates adipocyte inflammation by inhibiting NLRP3 pathway

**DOI:** 10.1038/srep12676

**Published:** 2015-08-03

**Authors:** Xukai Wang, Gang He, Yan Peng, Weitian Zhong, Yan Wang, Bo Zhang

**Affiliations:** 1 Department of Cardiovascular Internal Medicine, Institute of Field Surgery, Daping Hospital, Third Military Medical University, Chongqing, China; 2 Department of Medical Genetics, College of Basic Medicine, Third Military Medical University, Chongqing, China

## Abstract

Insulin resistance (IR) is a common feature of Type II diabetes, metabolic disorders, hypertension and other vascular diseases. Recent studies showed that obesity-induced inflammation may be critical for IR. To investigate the anti-inflammatory effect of sodium butyrate (NaB) on obesity-induced inflammation, the db/db mice were intraperitoneally injected with NaB for 6 weeks. Glucose control was evaluated by glucose tolerance test (GTT) and insulin tolerance test (ITT). Adipose tissue was harvested for gene expression analysis. 3T3-L1 adipocytes were treated with Tnf-α to mimic the inflammatory state and gene expression was detected by realtime PCR and Western blotting. Our results showed that NaB treatment improved glucose control in db/db mice as determined by GTT and ITT tests. Gene expression analysis showed that NaB inhibited cytokines and immunological markers including CD68, Interferon-γ and Mcp in adipose tissues in db/db mice. Moreover, NaB inhibited cytokine releasing in 3T3-L1 adipocytes treated with TNF-α. Further analysis of inflammation pathway showed that NLRP3 was activated in db/db mice, which was efficiently inhibited by NaB treatment. Our data suggest that inhibition of obesity-induced inflammation alleviates IR, and NaB might be a potential anti-inflammatory agent for obesity.

Insulin resistance (IR) is a state in which the body does not respond appropriately to circulating insulin. Under this condition, the biologic effect of insulin is blunted. IR is characterized by reduced tissue insulin sensitivity that leads to increased insulin production via compensatory secretion. IR occurs in several insulin-responsive tissues, including the liver, muscles and adipose tissue. Recent studies show that IR is commonly associated with obesity, diabetes, hypertension and other cardiovascular diseases^1^,^2^,^3^.

To date, the mechanisms of IR are not fully elucidated. Most studies have been focused on molecular mechanisms related to abnormalities in the interaction between insulin and its receptors, and in the subsequent events associated with multiple physiologic or pathologic processes. These mechanisms include hypoxia, oxidative stress, ER stress, dysregulation of lipid homeostasis, and mitochondrial dysfunction^4^. In addition to these mechanisms, other mechanisms may also be involved in IR. It is widely accepted that obesity and obesity-induced inflammation play a key role in IR. Adipose tissue (AT) is the main site of lipid storage, which regulates systemic lipid homeostasis. In obesity, AT is markedly infiltrated by proinflammatory macrophages and other leukocytes that secrete proinflammatory cytokines and chemokines^5^,^6^,^7^. Moreover, AT itself can act as an endocrine organ that produces various hormones including leptin, adiponectin, resistin, and cytokines, such as TNF-α and IL-6^8^,^9^. Thus AT is considered to be the main inflammatory organ that mediates obesity-induced inflammation.

Many signaling pathways associated with the development of obesity-induced inflammation have been identified, including NF-κB pathway, JNK pathway and inflammasome pathway^10^,^11^. The inflammasome complex consists of several proteins such as NLRPs (NALPs), ASC and caspase-1. Among these components, NLRPs are the cytosolic receptors that are involved in innate immune recognition of pathogen as well as intracellular and extracellular damages. Fourteen NLRPs members have been identified in human genome. NLRP3 is the most extensively studied member and can be activated by various stimuli, including microbial products, environmental factors, and endogenous molecules^12^,^13^,^14^. Vandanmagsar *et al.* found that the ablation of NLRP3 in mice prevents obesity-induced inflammasome activation in fat depots and the liver as well as enhances insulin signaling^15^. Stienstra *et al.* also showed that mice deficient in NLRP3, apoptosis-associated speck-like protein, or caspase-1 were resistant to the development of high-fat diet-induced obesity^16^. It has also been shown that NLRP3 inflammasome was associated with insulin resistance and impaired glucose metabolism in human^17^. These studies reveal a critical function of the inflammasome in obesity and insulin resistance, and suggest that the inhibition of the inflammasome is a potential therapeutic strategy.

Small molecular inhibitors of histone deacetylase (HDACs) (termed as HDACis) can specifically inhibit the activity of HDACs^18^. Butyrate is a four-carbon short-chain fatty acid (SCFA), which is a typical HDACis to inhibit class I/II HDAC. Its anti-inflammatory properties are also shown in an increasing number of animal and cellular models of inflammatory diseases. For example, oral administration of sodium butyrate (NaB) attenuates mucosal lesion and inflammation of intestinal mucosa in mice treated with dextran sulphate^19^. Anti-inflammatory effects of sodium butyrate and other SCFA were also observed in microglial inflammatory response^20^. Other HDACis such as Trichostatin A (TSA) and Vorinostat (SAHA) are also shown to suppress LPS-induced mRNA expression of proinflammatory mediators in macrophages^21^. All these studies indicate that HDACis are strong anti-inflammatory agents. However, studies regarding their roles in AT are rare in literature. In this study, we aimed to determine if HDAC inhibition ameliorates AT inflammation and improves tissue insulin sensitivity within in in*tro* and *in vivo* systems. Our results showed that NaB inhibited inflammatory responses of AT through NLRP3 pathway and improved tissue insulin sensitivity.

## Results

### NaB improved glucose control in db/db mice

**Figure 1:**
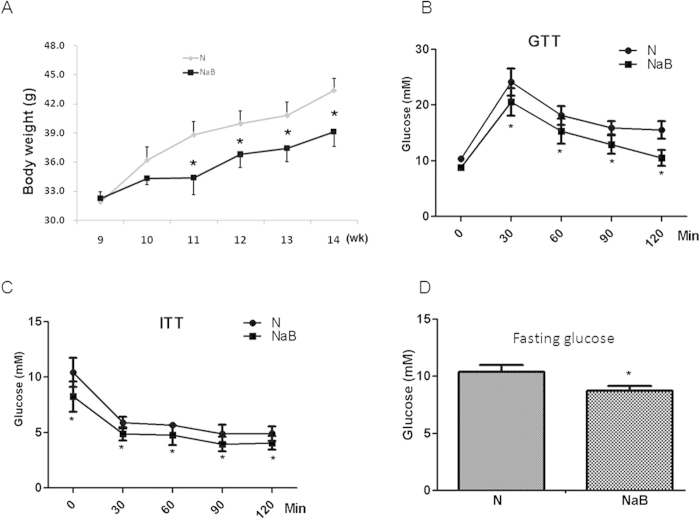
NaB improved glucose control in db/db mice. (**A**) NaB treatment significantly decreased the body weight of db/db mice. Intraperitoneal glucose tolerance test (**B**), insulin tolerance test (**C**) and fasting glucose (**D**) were detected at the end of treatment as described in Materials and Methods. Data are the means ± SE. *P < 0.05, by Student’s t test (N *vs* NaB). N: PBS-treated controls; NaB: NaB-treated group.

The db/db mice lacking of leptin receptor were used as an animal model of type 2 diabetes with obesity. NaB and TSA are classic HDACis that inhibit class I/II HDACs enzymes to cause histone hyperacetylation in many tissues and cell lines. In our study, NaB also induced histone protein H3 hyperacetylation in adipocyte cell line 3T3-L1 cells in a dose-dependent manner (Fig. S1A). To determine the therapeutic effects of NaB on diabetes, db/db mice were treated with or without NaB for 6 weeks. Mean body weight was significantly decreased by NaB treatment, compared with that of vehicle-treated controls (Fig. 1A). Glucose and insulin tolerance tests showed that NaB treatment ameliorated responses to glucose and insulin loading, compared with PBS-treated controls (Fig. 1B,C). Fasting glucose levels were also significantly decreased in NaB-treated mice (Fig. 1D). All these data suggest that NaB improves glucose control in db/db mice.

### NaB inhibited lymphocyte infiltration in adipose tissue

**Figure 2:**
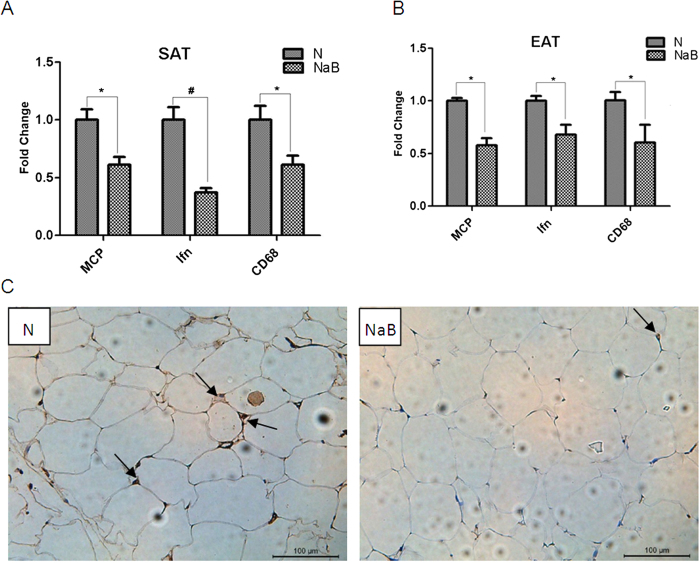
NaB inhibited lymphocyte infiltration into adipose tissues. (**A**–**B**) Epididymal adipose tissues (EAT) and subcutaneous adipose tissue (SAT) were harvested from db/db mice treated with or without NaB for six weeks. Total RNA was isolated for realtime RT-PCR. Data are expressed in ΔCt values normalized against the mean Ct of GAPDH. Fold changes were calculated as 2^(ΔΔCt_N-NaB_). *P < 0.05, ^#^*P* < 0.01 by Student’s t test. (**C**) Immunohistochemical staining of macrophage marker F4/80 in mouse EAT. N: Sham-treated group; NaB: NaB-treated group.

Recent studies show that obesity changes the functions of various types of immune cells, which play important roles in adipocyte inflammation. CD68, interferon-γ (Ifn), and monocyte chemoattractant protein-1 (MCP-1) were typical markers of immune cells including macrophages and other monocytes. In inflammation, the mRNA expression levels of these genes were upregulated by various stimuli. Quantitative RT-PCR analysis of the abundance of the mRNA of these genes confirmed the inflammation of AT in db/db mice, compared with those of age-matched WT mice (Fig. S2). NaB treatment significantly decreased the mRNA levels of these genes in both epididymal adipose tissue (EAT) and subcutaneous adipose tissue (SAT) in db/db mice, compared with those in vehicle-treated mice (Fig. 2A,B). To further confirm the inhibition of macrophage infiltration into AT in db/db mice by NaB, the protein marker of F4/80 was measured by immunostaining in EAT tissues. As shown in Fig. 2C, NaB treatment reduced the number of F4/80 positive cells, compared with vehicle-treated mice. These data clearly indicate that NaB inhibits lymphocyte inflation into adipose tissues in db/db mice.

### NaB inhibited cytokine gene expression in adipose tissues

**Figure 3:**
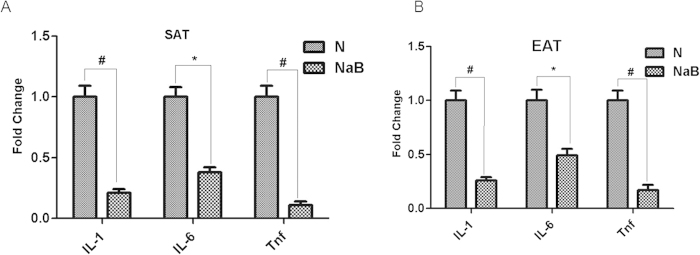
NaB inhibited cytokine expression in db/db mice adipose tissues. Epididymal adipose tissues (**A**) and subcutaneous adipose tissue (**B**) were harvested from db/db mice treated with or without NaB for six weeks. Total RNA was isolated for realtime RT-PCR. Data are expressed in ΔCt values normalized against the mean Ct of GAPDH. Fold changes were calculated as 2^(ΔΔCt_N-NaB_). *P < 0.05, ^#^*P* < 0.01 by Student’s t-test. N: PBS-treated group; NaB: NaB-treated group.

The literature suggests that adipose tissue is an important source of cytokines and chemokines. Therefore, we determined if NaB was able to decrease the expression of cytokines and chemokines in adipose tissue in diabetic mice. Db/db mice were treated with NaB or vehicle for six weeks and EAT and SAT were harvested for quantitative realtime RT-PCR analysis. Compared with normal phenotype mice (wild type), db/db mice had significantly increased inflammatory markers including IL-1, IL-6 and TNF-α (Fig. S3), indicating sustained inflammation in AT of db/db mice. However, these inflammatory markers were significantly decreased in both EAT and SAT of db/db mice by NaB treatment, compared with those in vehicle-treated mice (Fig. 3A,B).

### NaB reduced cytokine expression in 3T3-L1 cells treated by TNF-α

**Figure 4:**
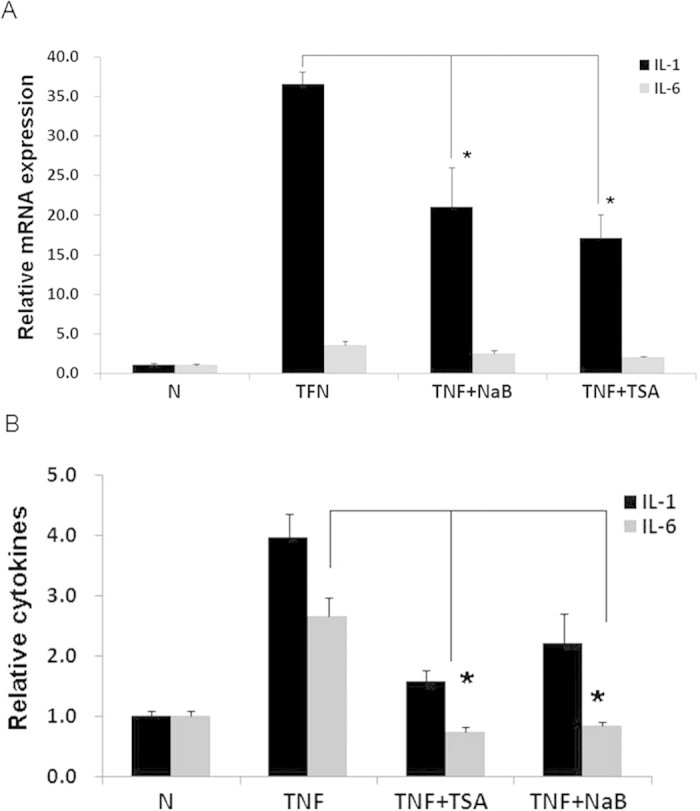
HDACi inhibited the cytokine expression in 3T3-L1 adipocytes. 3T3-L1 cells were treated with TNF-α (50 ng/mL) combined with or without NaB(1 mM) or TSA(0.5 μM) for 8 hr. Total RNA was isolated for realtime RT-PCR to detect the gene expression of IL-1β and IL-6 (**A**). Supernatants of cell culture were harvested for ELISA assay to detect IL-1 and IL-6 in the culture medium (**B**). N: Normal control. *P < 0.05 by t-test (TNF *vs* NaB or TSA).

To determine if NaB reduces cytokine production in adipose tissue by decreasing the gene expression of cytokines in adipocytes, NaB was used to treat 3T3-L1adipocytes that were pretreated with TNF-α to mimic the inflammatory state of AT. As expected, TNF-α treatment increased the mRNA levels of cytokines including IL-1α and IL-6 in a dose- and time-dependent manner in 3T3-L1 cells (Fig. S4). However, the effect of TNF-α was significantly attenuated by NaB or TSA (Fig. 4A). Moreover, the protein levels of IL-1α and IL-6 in the culture medium of 3T3-L1 cells treated with TNF-α were also decreased by NaB or TSA co-treatment (Fig. 4B). These results showed that HDACis inhibited cytokines gene expression in 3T3-L1 cells.

### HDACi inhibited activation of NLRP3 inflammasome in 3T3-L1 cells

**Figure 5:**
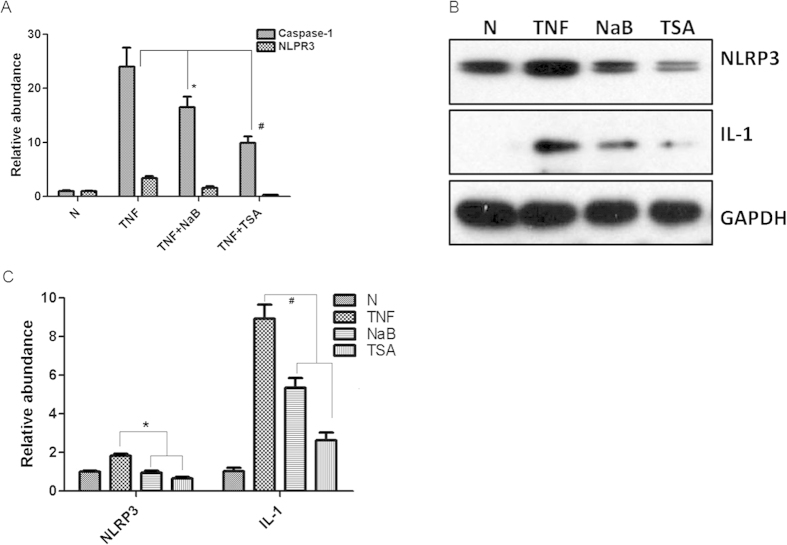
HDACi inhibited NLRP3 inflammasome pathway in 3T3-L1 cells. 3T3-L1 cells were treated with TNF-α combined without or with NaB (1 mM) and TSA (0.5 μM) for 8 hr. (**A**) Total RNA was isolated for realtime RT-PCR. mRNA expression was normalized to that of GAPDH. (**B**) Total proteins were isolated from treated 3T3-L1 cells for Western blots to detect the protein level of NLRP3 and IL-1. GAPDH was used as controls for equal loading. N: Normal control. (**C**) Densitometry measurement of Western blots. The experiments were repeated at least three times. *P < 0.05, ^#^*P* < 0.01 by t-test (TNF *vs* NaB or TSA).

It has been reported that inflammasome pathway is involved in obesity-induced inflammation. The main function of the inflammasome is to induce the maturation of IL-1β and IL-18 in response to danger signals. Here, we focused on NLRP3 inflammasome pathway, which is the most extensively studied one and has been found to be activated by various stimuli. TNF-α treatment increased the expression of NLRP3 and caspase-1 in a dose- and time-dependent manner in 3T3-L1 cells (Fig. 5S). As expected, the upregulation of NLRP3 and caspase-1 induced by TNF-α was attenuated by NaB treatment in 3T3-L1 cells (Fig. 5A). Moreover, the protein levels of NLRP3 and the maturation form of IL-1β were also reduced by NaB and TSA (another HDACi) (Fig. 5B,C). These results showed that HDACi inhibited the activation of NLRP3 inflammasome *in vitro*.

### NaB inhibited NLRP3 activation *in vivo*

**Figure 6:**
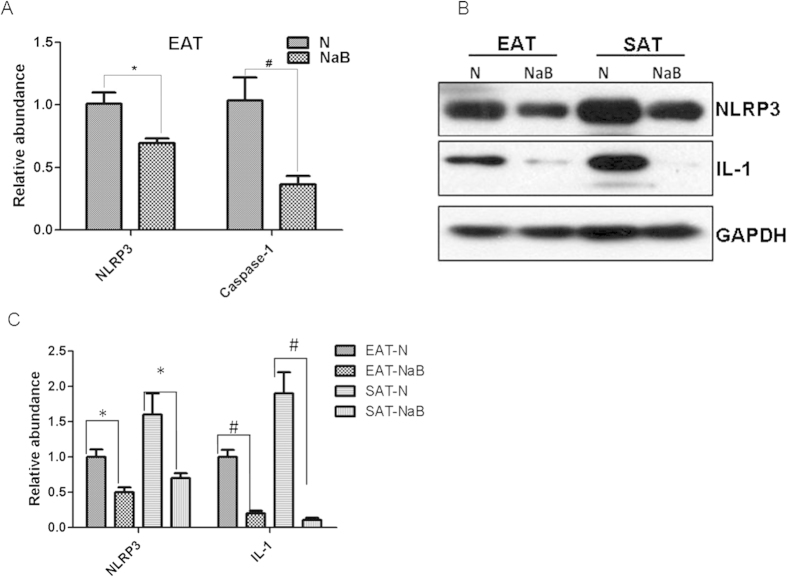
NaB inhibited LRP3 inflammasome pathway in db/db mice. Epididymal adipose tissues (EAT) and subcutaneous adipose tissue (SAT) were harvested from db/db mice treated with or without NaB for six weeks. Total RNA was isolated for realtime RT-PCR (**A**). Data are expressed in ΔCt values normalized against the mean Ct of GAPDH. Fold changes were calculated as 2^(ΔΔCt_N-NaB_). Total proteins were isolated for Western-blot (**B**). The bar graph of the densitometry measurement showed the decreased expression of NLRP3 and active IL-1 in db/db adipose tissues treated without or with NaB (**C**). *P < 0.05, ^#^*P* < 0.01 by Student’s t test.

To confirm that NaB also inhibits NLRP3 activation *in vivo*, AT tissues from db/db mice treated with NaB or vehicle were also analyzed. NLRP3 pathway was clearly activated in db/db mice as indicated by upregulation of mRNAs of NLRP3 and its effector caspase-1, compared with age-match WT mice (Fig. S6). After the treatment with NaB for six weeks in db/db mice, the mRNA abundance of NLRP3 and caspase-1 was significantly decreased, as determined by realtime RT-PCR (Fig. 6A). Moreover, the protein levels of NLRP3 and IL-1β were also decreased by NaB treatment in both SAT and EAT tissues of db/db mice (Fig. 6B,C). These data indicate that the NLRP3 pathway was activated in obese AT, which can be inhibited by NaB treatment.

## Discussions

Recent studies in obesity show that chronic low-grade inflammation is one of the characters found in expanding adipose tissue. Obesity and obesity-induced inflammation are thought to be key causes of IR. Besides functioning as the main site of lipid storage, AT can also express high levels of many inflammatory mediators involved in obesity-induced inflammation. Both adipocytes and immune cells contribute to the obesity-induced inflammation. In this study, NaB was applied *in vitro* and *in vivo* to detect if NaB has an anti-inflammatory effect in adipocytes and if this effect alleviates IR in obesity. Our results showed that NaB improved glucose control in obese db/db mice probably by reducing inflammatory cell infiltration into adipose tissues and inhibiting cytokine releasing from adipocytes. *In vitro*, NaB decreases inflammatory cytokines released by adipocytes (3T3-L1 cells) treated with TNF-α, probably by inhibiting the NLRP3 inflammasome signaling pathway. *In vivo*, NaB preventes NLRP3 pathway from activation in db/db mice.

Adipose tissue was considered to be an inflammatory organ in obesity. In our study, we showed that the expression of typical markers of immune cells was much higher in the adipose tissue of db/db mice than that of WT mice (Fig. S2). This suggested that increased immune cells including macrophage and other monocytes infiltrate into AT. Recent studies show that adipocytes also function as inflammatory cells^22^. Many molecular signaling pathways mediate the development of obesity-induced inflammation in AT. The roles of IKKβ/NFκB pathway and JNK pathway have been confirmed in other models. For example, NFκB activity is increased in obese animals^23^. When the IKKβ/NFκB pathway is inhibited by pharmacological inhibitors of IKKβ, the obesity-induced insulin resistance was improved in skeletal muscle^24^. Recent studies show that inflammasome pathway was involved in obesity and insulin resistance^25^. In this study, we explored the NLRP3 pathway, the most extensively studied inflammasome pathway. When activated, NLRP3 forms a complex with its adaptor ASC, which in turn promotes the recruitment of pro-caspase-1. After pro-caspase-1 was cleaved and activated by inflammasome complex, the cytosolic precursors of cytokines IL-1β and IL-18 were then processed by the active caspase-1 and these active cytokines were released. As these proinflammatory cytokines have been linked to the development of IR, NLRP3 pathway is thought to be involved in IR. For example, NLRP3 inflammasome activity and secretion of IL-1β were increased in obese individuals with metabolic disorders^26^. Also, NLRP3 pathway was activated by high-fat diet in mice^27^. Moreover, ablation of NLRP3 in mice prevents obesity-induced inflammasome activation and the development of IR^16^. Consistent with these findings, our study showed that the expression of NLRP3 in 3T3-L1 cells was greatly enhanced by TNF-α, which mimic the inflammatory state of AT. The products of NLRP3 pathway (IL-1β and IL-8) were also increased by TNF-α treatment in 3T3-L1 cells (Fig. 5). Data from obese mouse model also confirmed the activation of NLRP3 inflammasome pathway (Fig. 6).

HDACs are chromatin modifying enzymes known to play pivotal roles as transcriptional suppressors^28^. Recently, the roles of HDACs in obesity and metabolism have been extensively investigated in various models^29^. It has been reported that HDACs can regulate adipocyte differentiation and adipogenesis. For example, HDAC9 was found to be a negative regulator of adipogenic differentiation^30^. In these models, HDACs can function in regulation of transcription factors and signal transduction pathways. As specific inhibitor of HDACs, small chemical HDACi like NaB can efficiently suppress the activity of HDACs. In this study, we focused on the anti-inflammatory effect of NaB in adipose tissue. We found that the enhanced inflammation in adipose tissue can be efficiently reversed by NaB, which has been extensively investigated for cancer treatment, as well as other diseases such as diabetes, cystic fibrosis and obesity^31^,^32^,^33^,^34^. The protective effects of NaB are thought to be associated with cell cycle control, induction of apoptosis and selectively altering gene expression. Previous studies have also verified the anti-inflammatory property of butyrate in leukocytes, which may play an important role in obesity and obesity-induced inflammation^35^. In this study, we showed that butyrate treatment is able to reduce AT inflammation and improve glucose control in db/db mice, a well established model of obesity.

A slight but significant reduction of body weight was observed after NaB treatment. Similar effect of tributyrin (a butyrate prodrug) was also described in HFD-induced obesity mouse model^36^, as well as in other models within which diets were supplemented with butyrate or high contents of fibers^37^,^38^. Previous study also showed that NaB supplementation increased energy expenditure in HFD-induced obesity in C57BL/6 mice^37^. Moreover, modifications in adipose inflammation altered energy expenditure^39^. The effect on body weight of NaB treatment might be related to the increase in energy expenditure and oxygen consumption in these animals. In a mouse model with NaB-supplemented diet, the effect on body weight might be associated with the increased mitochondrial respiration at cellular level^37^. It was also showed that NaB supplementation did not reduce food intake, fat absorption or locomotor activity^37^. Thus, we speculate that the biological functions of NaB in AT include both the anti-inflammation effect and the influence of energy expenditure.

NaB is also a typical HDAC inhibitor, which inhibits class I/II HDAC enzymes. Histone hyperacetylation induced by NaB has been confirmed in many previous studies and this study (Fig. S1). Although studies have linked the HDAC inhibition activity of NaB to its anti-inflammation effect^40^,^41^, the exact mechanism of the anti-inflammation property of NaB is still unclear. Our study showed that NaB inhibited the expression of NLRP3 and pro-caspase 1, which could be one of the mechanisms. Interestingly, recent studies showed that other mechanisms were found to interpret the function of SCFA like NaB on energy metabolism based on G-protein coupled receptors (GPCR). Miletta MC *et al.* found that NaB increased intracellular calcium levels and enhanced growth hormone release from rat anterior pituitary cells via GPR41 and 43^42^. Moreover, the interaction of SCFA and GPR43 modulated colitis by regulating inflammatory cytokine production in mouse mononuclear cells^43^. All these data suggest that NaB play versatile roles via different molecular mechanism.

In conclusion, obesity in db/db mice is accompanied by the development of a chronic low-grade inflammation in adipose tissue, and NaB is able to slow the progression by inhibiting cytokine release and macrophages infiltration, and therefore helps to improve glucose control. The inflammation response in AT was at least partially mediated by NLRP3 pathway, which can be efficiently inhibited by NaB. All of these data support a possible role of butyrate as a potential anti-inflammatory agent for obesity.

## Methods

### Materials

TSA and NaB were obtained from Merck (Darmstadt, Germany). Primary antibodies against F4/80, NLRP3, IL-1β and GAPDH were purchased from Cell Signaling Technology (Beverly, USA). Trizol and reverse transcript kit was purchased from Invitrogen (Beijing, China). Tumor necrosis factor-α (TNF-α) was from Life Technologies (Carlsbad, USA), and IL-1β (CSB-E08054m) and IL-6(CSB-E04639m) ELISA kits were obtained from Cusabio Biotech Co. (Wuhan, China).

### Cell culture and HDACis treatment

3T3-L1 adipocyte cell line was obtained from Shanghai Cell Bank (http://www.cellbank.org.cn), and cultured in Dulbecco’s Modified Eagle’s Medium (DMEM) with 10% fetal bovine serum. Cells were treated with various concentrations of TNF-α and combined with NaB or TSA and then further cultivated for the indicated periods.

### Animals and treatments

All animal work was approved by the Animal Care and Use Committee of the Third Military Medical University, and the methods were carried out in accordance with the approved guidelines. C57BL/6J and C57BL/6J db/+ mice were obtained from The Jackson Laboratory (Bar Harbor, ME). Mice were housed in a room with controlled temperature and 12 hr light-dark cycle and had free access to water and standard chow. Eight-week-old male db/db mice (n = 16) were randomly divided in two groups: control group (N) and sodium butyrate group (NaB), which received intraperitoneal (ip) injection of saline or NaB (1.0 g/kg) every other day for 6 weeks, respectively. Administration of NaB was modified according to other study^44^. Body weights were recorded twice every week. At the end of the protocol, mice were euthanized by cervical dislocation for tissue harvesting.

### GTT and ITT

GTT was performed at the end of NaB treatment. After 12 hr of fasting (time 0), mice were *ip* injected with a 50% glucose solution (2.0 g/kg body weight). Blood samples were collected at 0, 30, 60, 90, and 120 min after glucose injection for determination of glycemia.

Intraperitoneal insulin tolerance test was performed by an i.p injection of insulin at a dose of 0.75 units/kg body weight in mice after 4 hours of fasting. Blood samples were collected at 0, 30, 60, 90, and 120 min after insulin injection for serum glucose determination.

### ELISA assay

IL-1 and IL-6 secreted by cultured 3T3-L1 cells in cell culture medium were determined with ELISA using commercial kits. Experiments were performed in accordance with the manufacturer’s instruction and each sample was analyzed in duplicate. Relative cytokine release was normalized to that of untreated controls.

### Realtime RT-PCR

An amount of 1 μg total RNA was reverse transcribed to cDNA according to the manufacturer’s directions (Roche (China) Ltd., Shanghai, China). Quantitative PCR amplification was performed with a 10 μL final reaction mixture consisting of 0.5 μL of reverse transcription reaction mixture, 0.1 μM of the each sense and antisense primer (Table S1) and 1 × PCR mixture of SsoAdvanced SYBR Green Supermix (Bio-Rad), using the Bio-Rad iCycler system (Bio-Rad). The PCR conditions were initial denaturation at 95 °C for 5 min followed by 40 cycles of PCR reaction: denaturation at 95 °C for 20 s; annealing at 58 °C for 30 s and elongation at 72 °C for 30 s. Gene expression was normalized to the corresponding GAPDH level and was presented as the fold difference relative to GAPDH gene expression.

### Immunohistochemical staining

Fresh tissues were collected and fixed in 10% formalin solution. Tissue slides were obtained through serial cross-section cutting in 8 μm thickness and processed with standard procedure. The slide was stained with a F4/80 antibody diluted at 1:200. After being washed, the slide was incubated with a biotinylated secondary antibody, and the color reaction was performed using ABC reagent (Zhongshan Co., Beijing, China).

### Western blotting

Freshly isolated tissues (EAT and SAT) and cell pellets harvested at indicated times were homogenized in ice-cold RIPA buffer containing complete protease inhibitors cocktail (Roche (China) Ltd., Shanghai, China). Total protein was resolved on SDS-PAGE and transferred onto a nitrocellulose membrane. After blocking in 3% nonfat milk for 30 min, membranes were incubated with antibody against total NLRP3, IL-1 and GAPDH, followed by incubation with corresponding secondary antibodies (Zhongshan Corp., Beijing, China). The bands were visualized by using the enhanced chemiluminescence system (Pierce, USA). The expression of individual protein was normalized to GAPDH.

### Statistical analysis

One-sample t-test was used to analyze the difference of the gene expression ratios of drug-treated versus normal samples. A *p*-value less than 0.05 was considered significant. Results were obtained from at least three separate experiments and expressed as means ± SEM.

## Additional Information

**How to cite this article**: Wang, X. *et al.* Sodium butyrate alleviates adipocyte inflammation by inhibiting NLRP3 pathway. *Sci. Rep.* **5**, 12676; doi: 10.1038/srep12676 (2015).

## Supplementary Material

Supplementary Information
