# Supplementary material for: Sodium butyrate alleviates adipocyte inflammation by inhibiting NLRP3 pathway
Source: Sci Rep. 2015 Aug 3;5:12676. doi: 10.1038/srep12676 (PMC4522654; doi:10.1038/srep12676)
Supplement: Supplementary Information [file srep12676-s1.pdf]

Sodium butyrate alleviates adipocyte inflammation by inhibiting NLRP3 pathway

Xukai Wang[1], Gang He[2], Yan Peng[1], Weitian Zhong[1], Yan Wang[2], Bo Zhang[2]

1 Department of Cardiovascular Internal Medicine, Institute of Field Surgery, Daping Hospital, Third Military Medical University, Chongqing, China

2 Department of Medical Genetics, College of Basic Medicine, Third Military Medical University, Chongqing, China

Corresponding author: Dr. Bo Zhang, Department of Medical Genetics, Third Military Medical University, Chongqing 400038, China. FAX: 86-23-65318610, TEL: 86-23-68753670, e-mail: bo_zhang@yahoo.com

Supplementary Materials

Table S1.Sequence of real-time RT-PCR primers

Gene Name	Sequence	Product (bp)
IL-1	F: GCTACCTGTGTCTTTCCCGT<br>R: CATCTCGGAGCCTGTAGTGC	164
IL-6	F: CAATGGCAATTCTGATTGTATG<br>R: AGGACTCTGGCTTTGTCTTTC	216
MCP-1	F: AGCACCAGCCAACTCTCACTG<br>R: CTCATTGGGATCATCTTGCTG	185
CD68	F: TACCCAATTCAGGGTGGAAG<br>R: CTCGGGCTCTGATGTAGGTC	191
Interferon	F: CTCTGAGACAATGAACGCTAC<br>R: TTCTTCCACATCTATGCCACT	148
Caspase-1	F: AACAGAACAAAGAAGATGGCACA<br>R: AGCTCCAACCCTCGGAGAAAG	196
NLRP3	F: TGAAGGACCCACAGTGTAACTT<br>R: CAAGGGCATTGCTTCGTAGAT	138
GAPDH	F: GGAGAGTGTTTCCTCGTCCC<br>R:ACTGTGCCGTTGAATTTGCC	202

F: forward primer; R: reverse primer.

Figure S1

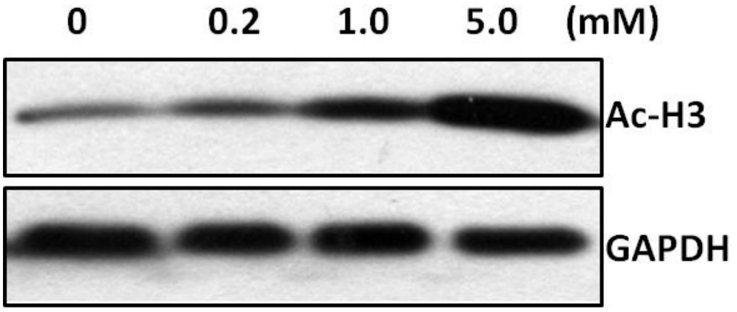

Figure S1 NaB treatment induced hyperacetylation of histone H3. 3T3-L1 cells were treated with increasing concentration of NaB for 24h. Total proteins were then isolated and theacetylation level of histone H3 was determined by western blot. GAPDH was used as internal control.

Figure S2

A

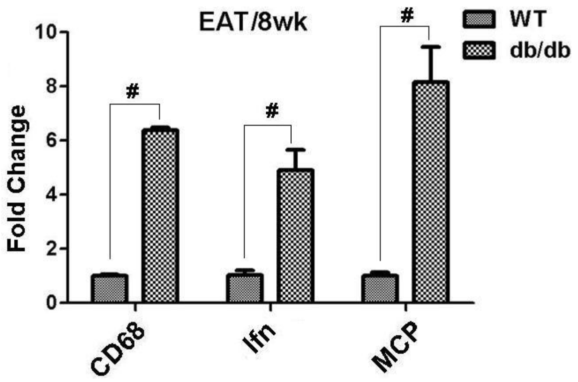

B

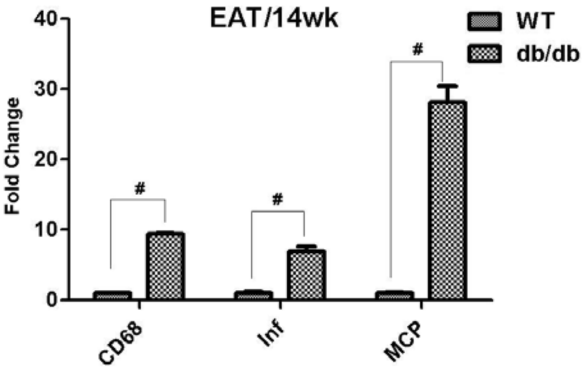

Figure S2 mRNA expression of CD68, Ifn and MCP-1 in control and db/db mice. Epididymal adipose tissues (EAT) were harvested from db/db mice and its wild type control at the age of 8 week (A) and 14 week (B). Total RNA was isolated for realtime RT-PCR. Data are expressed in ΔCT values normalized against the mean CT of GAPDH. # *P*< 0.01 by Student's t test.

Figure S3

A

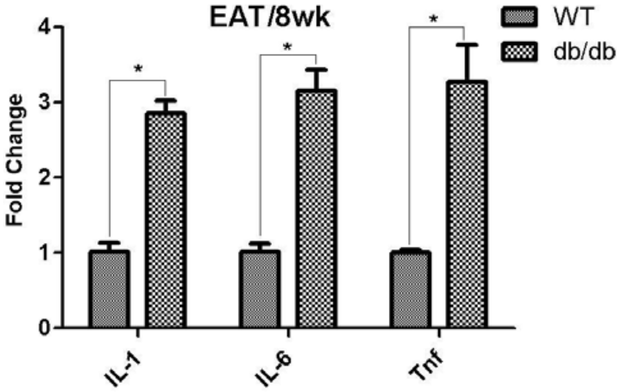

B

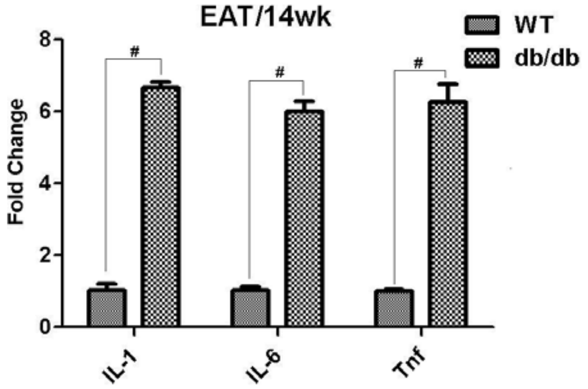

Figure S3 mRNA expression of IL-1, IL-6 and Tnf in control and db/db mouse EAT. Epididymal adipose tissues (EAT) were harvested from db/db mice and its wild type control at the ages of 8 and 14 week. Total RNA was isolated for realtime RT-PCR. Data are expressed in ΔCT values normalized against the mean CT of GAPDH. *P< 0.05, # P< 0.01 by Student’s t test.

Figure S4

Fig.S4A

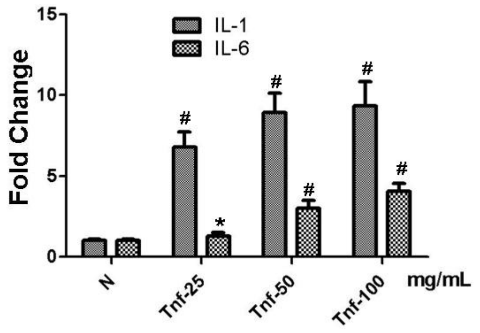

Fig.S4B

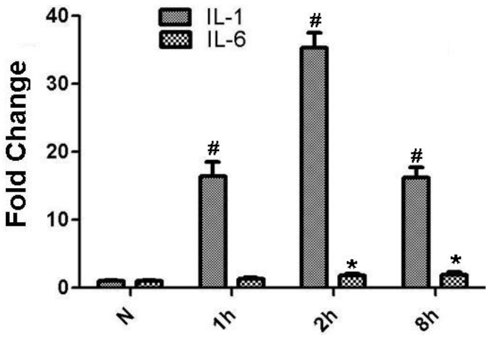

Figure S4 Effects of TNF-α on gene expression of cytokine in 3T3-L1 cells. (A) 3T3-L1 cells were treated with increasing concentration of TNF-α for 4 hours. (B) 3T3-L1 cells were treated with TNF-α (50ng/ml) for indicated periods. Total RNA was isolated for realtime RT-PCR. Data are expressed in ΔCT values normalized against the mean CT of GAPDH. *$P < 0.05$, # $P < 0.01$ by Student's t test.

Figure S5

Fig.S5A

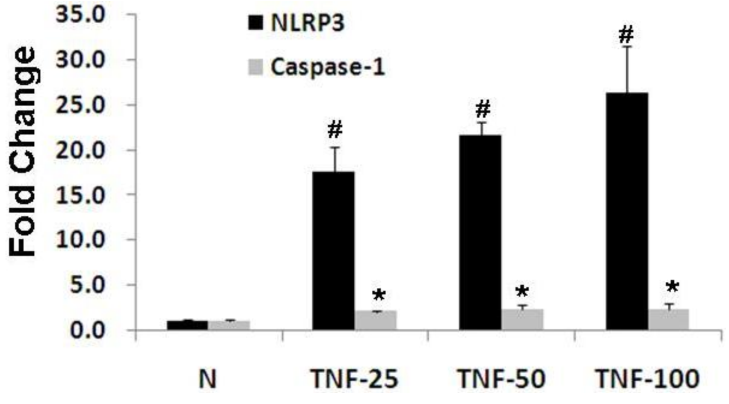

Fig.S5B

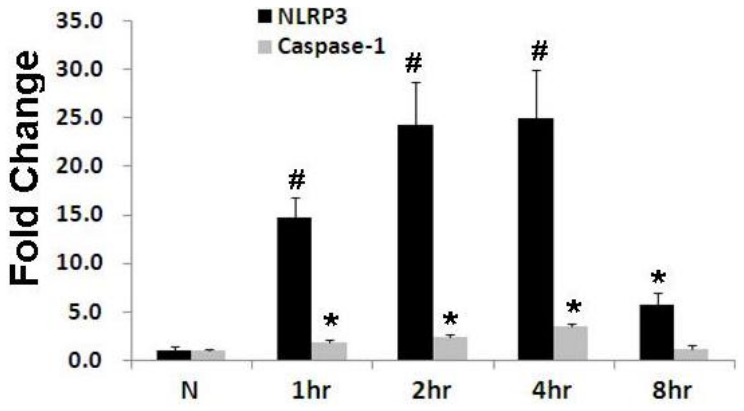

Figure S5 Effects of TNF-α on gene expression of NLRP3 and Caspase-1 in 3T3-L1 cells. (A) 3T3-L1 cells were treated with increasing concentration of TNF-α for 4hr. (B) 3T3-L1 cells were treated with TNF-α (50ng/ml) for indicated periods. Total RNA was isolated for realtime RT-PCR. Data are expressed in ΔCT values normalized against the mean CT of GAPDH. *P< 0.05, # P< 0.01 by Student's t test.

Figure S6

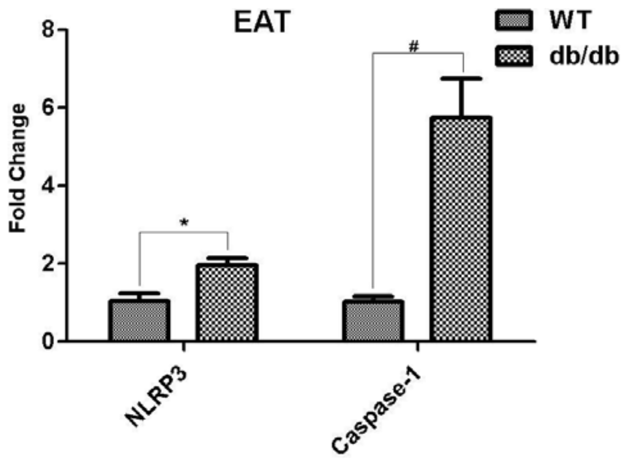

Figure 6 mRNAexpression of NLRP3 and Caspase-1 in mice. Epididymal adipose tissues (EAT) were harvested from Db/db mice and its wild type control. Total RNA was isolated for realtime RT-PCR. Data are expressed in ΔCT values normalized against the mean CT of GAPDH. *P< 0.05, # P< 0.01 by Student's t test.
